# Physiological, Transcriptome, and Metabolome Analyses Reveal the Tolerance to Cu Toxicity in Red Macroalgae *Gracilariopsis lemaneiformis*

**DOI:** 10.3390/ijms25094770

**Published:** 2024-04-27

**Authors:** Xiaojiao Chen, Yueyao Tang, Hao Zhang, Xiaoqian Zhang, Xue Sun, Xiaonan Zang, Nianjun Xu

**Affiliations:** 1 Key Laboratory of Marine Biotechnology of Zhejiang Province, School of Marine Sciences, Ningbo University, Ningbo 315211, China; chenxiaojiao@nbu.edu.cn (X.C.); tangyueyao98@126.com (Y.T.); zhanghao@nbu.edu.cn (H.Z.); zhangxiaoqian@nbu.edu.cn (X.Z.); sunxue@nbu.edu.cn (X.S.); 2 Key Laboratory of Marine Genetics and Breeding, Ministry of Education, Ocean University of China, Qingdao 266003, China; xnzang@ouc.edu.cn

**Keywords:** Cu toxicity, red macroalgae, enzymatic oxidation, *Gracilariopsis lemaneiformis*, secondary metabolite

## Abstract

Heavy metal copper (Cu) will inevitably impact the marine macroalgae *Gracilariopsis lemaneiformis* (*G. lemaneiformis*), which is a culture of economic importance along China’s coastline. In this study, the detoxification mechanism of Cu stress on *G. lemaneiformis* was revealed by assessing physiological indicators in conjunction with transcriptome and metabolome analyses at 1 d after Cu stress. Our findings revealed that 25 μM Cu stimulated ROS synthesis and led to the enzymatic oxidation of arachidonic acid residues. This process subsequently impeded *G. lemaneiformis* growth by suppressing photosynthesis, nitrogen metabolism, protein synthesis, etc. The entry of Cu ions into the algae was facilitated by ZIPs and IRT transporters, presenting as Cu^2+^. Furthermore, there was an up-regulation of Cu efflux transporters HMA5 and ABC family transporters to achieve compartmentation to mitigate the toxicity. The results revealed that *G. lemaneiformis* elevated the antioxidant enzyme superoxide dismutase and ascorbate-glutathione cycle to maintain ROS homeostasis. Additionally, metabolites such as flavonoids, 3-O-methylgallic acid, 3-hydroxy-4-keto-gama-carotene, and eicosapentaenoic acid were up-regulated compared with the control, indicating that they might play roles in response to Cu stress. In summary, this study offers a comprehensive insight into the detoxification mechanisms driving the responses of *G. lemaneiformis* to Cu exposure.

## 1. Introduction

Marine pollution is characterized as the introduction of substances or energy into the marine environment, primarily due to human activities that harm marine living resources, endanger human health, and disrupt fishing and other legal activities, thereby diminishing the value of seawater and reducing the quality of the marine environment. Heavy metals (HMs) are a prevalent source of marine pollution, noted for their high toxicity, environmental persistence, insidiousness, and bioaccumulation [1,2]. Copper (Cu) is an essential micronutrient which is involved in photosynthesis, nitrogen metabolism, and hormonal signaling pathways in plants [1]. However, excess Cu can interact with tyrosine residues located in the functional center of photosystem II, severely promoting the hydrolysis of the oxygen release complex and inhibiting the conversion of H_2_O to O_2_ [3,4]. Furthermore, it can also interfere with photosystem II by interacting with the non-heme iron and cytochrome complex. Numerous studies have demonstrated that an excess Cu can inactivate certain enzymes and proteins by supplanting their cofactors. For instance, the substitution of a magnesium ion (Mg^2+^) with Cu^2+^ in photosystem II leads to the impairment of the function of the antenna protein LHC II and the inhibition of photosynthesis [5]. Another significant effect of Cu excess is the induction of oxidative stress conditions, marked by the accumulation of reactive oxygen species (ROS) such as superoxide anions, hydroxyl radicals, and hydrogen peroxide (H_2_O_2_) [6,7]. While low levels of ROS can function as signaling molecules to induce gene expression, excessive ROS will cause oxidative stress and cellular damage. For example, excess ROS also attack the membrane lipids of chloroplasts and thylakoids, causing oxidative stress in plant cells and reducing the content of photosynthetic pigments and electron carriers, thereby inhibiting electron transport in photosynthesis [8].

Many mechanisms of plant tolerance to excessive Cu stress have been proposed. Plants can alleviate Cu toxicity by modulating the levels of transporter proteins such as Cu transporters (COPT), ferric reductase oxidase (FRO), heavy metal ATPase (HMA), and ATP-binding cassette (ABC)-type transporters to control the uptake and excretion of Cu [6,8]. They also regulate the synthesis of metal-binding proteins in metallothioneins (MTs) and phytochelatins (PCs) to enhance Cu sequestration into the vacuoles or Cu exclusion outside the cytoplasm, thereby inhibiting the overgeneration of ROS [9]. Another plant detoxification mechanism consisting of different types of antioxidant enzyme systems includes superoxide dismutase (SOD), catalase (CAT), glutathione peroxidase (GPx), L-ascorbate peroxidase (APX), and gluathione reductase (GR) [8,10]. Many studies have uncovered small soluble metabolites, including antioxidants such as glutathione (GSH), ascorbate acid (AsA), and flavonoids; organic acids such as malate, citrate, and oxalate; and fatty acids [11,12].

*Gracilariopsis lemaneiformis* (*G. lemaneiformis*) is the second most cultivated macroalgae in China, holding significant economic value. As a primary producer in marine ecosystems, *G. lemaneiformis* exhibits robust capabilities as bioremediator for copper-contaminated oceans [13,14]. The bioconcentration factors of Cu in *G. lemaneiformis* can reach 1000-fold [15]. This is achieved through the synthesis of various bioactive compounds, including polysaccharides and proteins, with carboxyl, hydroxyl, and amine functional groups, which bind to Cu [16,17]. Additionally, *G. lemaneiformis* serves as an excellent bait for aquatic species such as abalone and is a primary source of edible agar [16,18]. The accumulation of Cu in *G. lemaneiformis* can be transferred to humans via the food chain, thereby presenting potential health risks [19]. So far, studies have mainly focused on the effects of Cu on growth and the accumulation of Cu in *G. lemaneiformis*. Surprisingly, even though *G. lemaneiformis* is a widely cultivated macroalgae in coastal regions, its mechanisms of Cu tolerance are still not clear.

To evaluate the Cu transporter, detoxification, molecular regulatory networks, and key metabolic pathways to elucidate the tolerance mechanism of Cu toxicity in *G. lemaneiformis*, we employed physiological, transcriptome, and metabolome analyses. In the current study, the effect of Cu on photosynthetic pigment content, chlorophyll fluorescence parameters, nitrate reductase activity, and H_2_O_2_ and flavonoid content were measured after Cu stress. Then, we conducted a comparative transcriptome and metabolome analysis to investigate the genes and metabolic pathways responsive to Cu.

## 2. Results and Discussion

### 2.1. Photosynthesis and Nitrogen Metabolism

**Figure 1:**
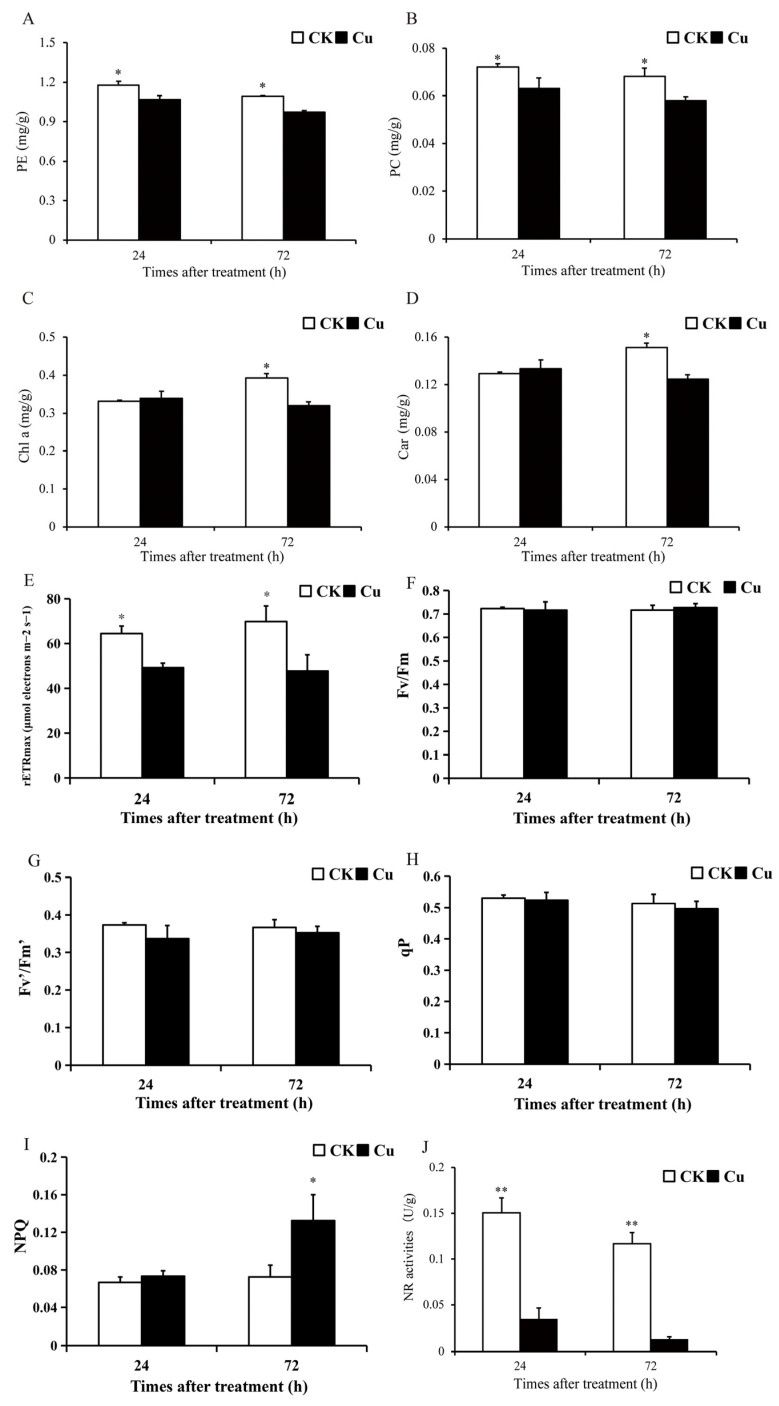
An overview of photosynthesis and nitrate reductase changes of *G. lemaneiformis* under Cu stress. (**A**) Phycoerythrin (PE) content, (**B**) phycocyanin (PC) content, (**C**) chlorophyll a (Chl a) content, (**D**) carotenoid (Car) content, (**E**) maximum photosynthetic electron transfer rate (rETRmax), (**F**) maximum efficiency of PSII (Fv/Fm), (**G**) effective photochemical efficiency of PSII (Fv′/Fm′), (**H**) photochemical quenching (qP), (**I**) mon-photochemical quenching (NPQ), and (**J**) activities of nitrate reductase (NR). Asterisks (*) and (**) indicate significant differences (*p* < 0.05 and *p* < 0.01, respectively). The number of replicates *n* = 3 and means are shown ± SD.

Photosynthesis is an important link in the conversion of CO_2_ to carbohydrates in plants, which is the basis of plant growth and development and one of the most sensitive physiological processes in plants under abiotic stress [20]. Photosynthetic pigment content and chlorophyll fluorescence parameters serve as prevalent indicators of photosynthesis levels in algae. The contents of phycoerythrin (PE), phycocyanin (PC), chlorophyll a (Chl a), and Car in *G. lemaneiformis* were measured after 24 and 72 h of Cu treatment, with the findings illustrated in Figure 1A–D. Notably, the photosynthetic pigment content exhibited a significant decrease in the Cu-treated group compared to the CK group. Specifically, PE and PC contents diminished by 9.4–11.2% (*p* < 0.05) and 12.5–15.0% (*p* < 0.05) at 24 and 72 h, respectively. Chl a and Car contents declined by 18.3% (*p* = 0.03) and 17.7% (*p* = 0.02) at 72 h, respectively. The findings revealed a significant reduction in the contents of PE, PC, Chl a, and Car in the group treated with Cu, suggesting that Cu stress disrupted the photosynthetic system of *G. lemaneiformis.* This observation aligns with previous research conducted by Brown et al. (2012) [21].

Chlorophyll fluorescence parameters were also determined after 24 and 72 h of treatment, and Cu treatment reduced the maximum photosynthetic electron transfer rate (rETRmax) of *G. lemaneiformis* by 23.5–31.6% (*p* < 0.05) (Figure 1E). However, Cu reduced the maximum efficiency of PSII (Fv/Fm), the effective photochemical efficiency of PSII (Fv′/Fm′), and photochemical quenching (qP) to some extent, but there was no significant difference between the groups (Figure 1F–H). In addition, Cu stress enhanced non-photochemical quenching (NPQ) at 72 h by 82.8% (*p* = 0.001) (Figure 1I). The non-photochemical quenching aspect reflects the plants’ ability to thermally dissipate excess light energy into heat energy, thereby providing a form of resistance against adversity [22,23]. Our study demonstrated that Cu reduced rETRmax but increased NPQ in *G. lemaneiformis*, suggesting that *G. lemaneiformis* may resist Cu stress by enhancing the photoprotective capacity of the photosystem.

Nitrogen metabolism, which provides the nitrogen backbone for protein synthesis, is a complex process involving multiple steps such as NO_3_^−^ transport, NO_3_^−^ reduction, NO_2_^−^ reduction, and NH_4_^+^ incorporation [24]. The first and rate-limiting step in this process is NR-catalyzed nitrogen reduction. Nitrate reductase (NR) is the key enzyme in the conversion of nitrate to nitrite during nitrogen metabolism. High levels of HM stress can disrupt plant nitrogen metabolism [25]. After 24 and 72 h of Cu treatment, NR activity was significantly reduced by 76.8% (*p* = 0.003) and 88.5% (*p* = 0.002) (Figure 1G). These results suggest that Cu stress has reduced the nitrogen assimilation efficiency of *G. lemaneiformis*. A similar effect was observed in rice, where Cu stress reduced the enzyme activities of NR and GS, leading to a reduction in plant biomass [26].

### 2.2. Content of H_2_O_2_ and Flavonoids

**Figure 2:**
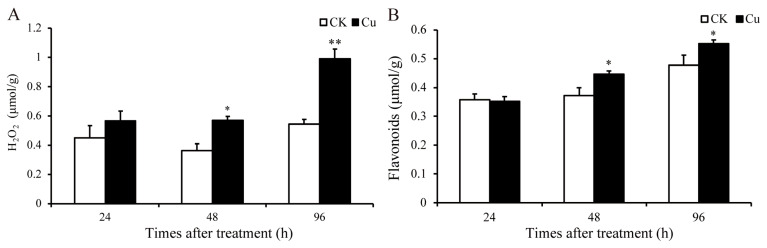
Changes in content of H_2_O_2_ and flavonoids in *G. lemaneiformis* under Cu stress. (**A**) H_2_O_2_ and (**B**) flavonoids. Asterisks (*) and (**) indicate significant differences (*p* < 0.05 and *p* < 0.01, respectively). The number of replicates *n* = 3 and means are shown ± SD.

Research has demonstrated that HM stress can stimulate a significant production of ROS [6]. H_2_O_2_ is the main component of ROS, and the content of H_2_O_2_ was quantified at 24, 48, and 96 h in this study. As depicted in Figure 2A, the H_2_O_2_ level increased by 57.5% (*p* = 0.02) and 82.5% (*p* = 0.005) at 48 and 96 h, respectively. The notable increase in the H_2_O_2_ level indicates that Cu stress has promoted the accumulation of ROS in *G. lemaneiformis*. The primary pathway for ROS-induced tissue damage is believed to be peroxidation, with MDA serving as the principal marker of lipid oxidative damage [27]. Moreover, Cu promoted the accumulation of MDA in *G. lemaneiformis* [9].

Flavonoids, non-enzymatic antioxidants prevalent in plants, serve to scavenge ROS. The content of these compounds was quantified at 24, 48, and 96 h. As depicted in Figure 2, the H_2_O_2_ content increased by 57.5% (*p* = 0.02) and 82.5% (*p* = 0.005), and flavonoids increased by 20.1% (*p* = 0.03) and 15.6% (*p* = 0.04) at 48 and 96 h, respectively. In our prior studies, we observed that the contents of GSH and AsA, along with the activity levels of SOD and APX within the antioxidant system, were also augmented by Cu [9]. These results indicated that Cu stress has promoted the production of ROS, and the antioxidant system might be involved in the detoxification and tolerance mechanisms against Cu in *G. lemaneiformis*.

### 2.3. Identification and Validation of Differentially Expressed Genes

To further investigate the molecular mechanism underlying Cu detoxification and tolerance in *G. lemaneiformis*, we conducted a transcriptome analysis at 24 h after Cu treatment. In total, 2075 DEGs including 935 up-regulated and 1140 down-regulated ones were obtained, respectively. The results of qRT-PCR were consistent with those of the transcriptome analysis (Supplementary Figure S1), indicating that our transcriptome data are reliable.

### 2.4. GO and KEGG Enrichment Analyses

To elucidate the roles of DEGs, GO and KEGG enrichment analyses were performed on the down-regulated and up-regulated DEGs, respectively. The GO analysis revealed that the down-regulated DEGs were significantly enriched in structural molecule activity, ribosomes, translation, the peptide/amide biosynthetic process, the peptide metabolic process, photosynthesis, and gene expression (Supplementary Figure S2B). Furthermore, the KEGG enrichment analysis indicated that these down-regulated DEGs were associated with various biological processes including translation and energy metabolism, including photosynthesis and nitrogen metabolism, carbohydrate and amino acid metabolism, etc. (Table 1).

The GO analysis showed that the up-regulated DEGs were significantly enriched in ADP binding, cell cycles, adenyl/purine nucleotide binding, carbohydrate derivative binding, and chromosome segregation (Supplementary Figure S2A). As for KEGG enrichment, the up-regulated DEGs were related to metabolism including lipid and carbohydrate metabolism, secondary metabolite biosynthesis and metabolism, ABC membrane transport, basal transcription factors, and DNA replication (Table 1). These findings indicated that Cu stress has a positive effect on membrane transport, lipid metabolism, carbohydrate metabolism, and genetic information processing in *G. lemaneiformis*.

### 2.5. Specific DEGs in Response to Cu

#### 2.5.1. DEGs Involved in Cu and Other Element Transporters

Cu is one of the essential metal elements for plant growth and development, which is absorbed from the environment through copper transport proteins including ferric reductase oxidase (FRO), Cu transporters (COPT), heavy metal ATPase (HMA), and zinc/iron transporters (ZIP/IRT) [28]. Partial Cu^2+^ is reduced to Cu^+^ by FRO at the cell surface [29] and the reduced Cu^+^ ion crosses the plasma membrane through the high-affinity transporter COPT and enters the cell [30]. However, FRO and COPT were not differentially expressed under Cu stress in *G. lemaneiformis*. Additionally, Cu^2+^ can also permeate the plasma membrane via ZIP and IRT transporters [28]. Our results showed that the expression of genes encoding ZIP1, ZIP3, ZIP6, and IRT1 was significantly up-regulated under Cu stress (Table 2). Cu significantly up-regulated Cu^2+^ transporter genes encoding ZIP1, ZIP3, ZIP6, and IRT1 rather than FRO and COPT, suggesting that Cu^2+^ may be the main form of Cu ions in *G. lemaneiformis.*

The Cu ion that enters the cell is partially involved in growth metabolism as a cofactor for enzymes such as SOD [1] and plastocyanin, or acts as a cofactor to facilitate the binding of ethylene to its receptor [31]. However, excess Cu becomes an efflux from the extracellular matrix or combines with phytochelatins (PCs) and is transported to the vacuole, Golgi apparatus, and other organelles mediated by HMA5 proteins for detoxification [32]. PCs are generated by the polymerization of GSH, both of which can chelate heavy metals because of the high affinity of metal ions for the sulfhydryl groups in these compounds. In our previous study, the GSH content of *G. lemaneiformis* was highly increased under Cu stress [9]. In addition, Song et al. (2013) [33] reported that ATP-binding cassette (ABC)-type transporters also play a role in transferring Cu-PCs to the vacuole. The expression of a gene encoding HMA5 and eight genes encoding ABC transporters was significantly up-regulated in response to Cu stress (Table 2). The above results suggest that excess Cu led to the increased uptake of Cu by transporters in *G. lemaneiformis*, and then enhanced Cu sequestration and efflux by overexpressing sulfur-containing chelating agents and efflux transporters to avoid cellular damage from excess Cu.

Notably, the expression of other element transporters, including P, Mg, and N transporters, was down-regulated by Cu (Table 2), suggesting that excess Cu could potentially reduce the availability of P, Mg, and N.

#### 2.5.2. DEGs Involved in Antioxidant System

As shown in Table 3, the up-regulated DEGs were enriched to peroxisome, of which 12 of 15 were significantly enhanced by 2–74-fold. One gene encoding D-amino acid oxidase (DAO), which catalyzes D-amino acid and oxygen to H_2_O_2_ [34], was induced by Cu. Meanwhile, two genes encoding MPV17, which negatively regulate H_2_O_2_ biosynthesis [35], showed a down-regulation, suggesting that Cu promoted ROS biosynthesis. Furthermore, Cu-induced peroxin-10, peroxin-13, and ABCD3 were beneficial to the synthesis of peroxisome and the antioxidant enzyme superoxide dismutase (SOD), which reduces ROS.

In addition, DEGs encoding enzymes associated with the GSH-AsA cycle played an important role in REDOX, such as APX, glutathione reductase (GR), and glutathione-S-transferase (GST), and were also significantly up-regulated by 2–2.6-fold (Table 3). The antioxidants and antioxidant enzymes involved in the GSH-AsA cycle effectively scavenged excessive intracellular reactive oxygen species, thereby mitigating oxidative damage caused by Cu stress. Concurrently, GSH serves as a precursor for PC synthesis, and the enzyme GST, which is pivotal in the glutathione-binding reaction that initiates PC synthesis, is frequently overexpressed in plants under heavy metal stress [36,37]. GST expression was markedly induced by Cu in *G. lemaneiformis*, potentially signifying enhanced PC synthesis. Furthermore, genes encoding peroxidase-related enzymes such as SOD, EPHX, and PEX were significantly up-regulated under Cu stress. In our prior studies, we observed a notable increase in GSH and AsA contents under Cu stress [9]. Consequently, increasing enzyme activity, synthesizing a large amount of compounds that scavenge reactive oxygen species, and up-regulating the expression of antioxidant enzyme gene expression may represent viable strategies to mitigate oxidative damage and cope with Cu stress in *G. lemaneiformis*.

#### 2.5.3. DEGs Involved in Lipid Metabolism and Betalain Biosynthesis

**Figure 3:**
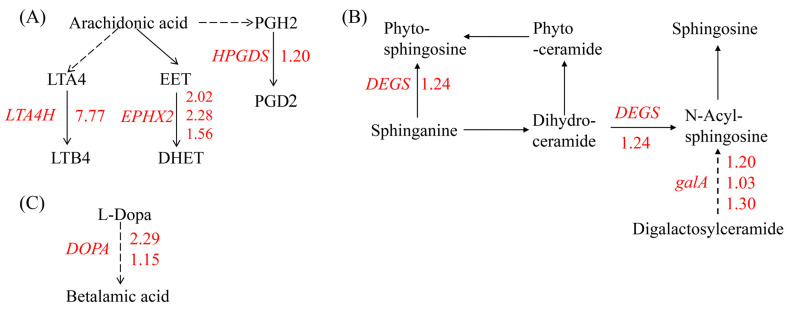
An overview of DEGs involved in lipid metabolism and betalain biosynthesis. (**A**) Arachidonic acid metabolism, (**B**) sphingolipid metabolism, and (**C**) betalain biosynthesis. The number next to the gene represents log_2_ (fold change) and the red number represents the up-regulation expression of genes. GENE: *LTA4H*: gene encoding leukotriene-A4 hydrolase, *EPHX2*: gene encoding soluble epoxide hydrolase, *HPGDS*: gene encoding prostaglandin-H2 D-isomerase, *DEGS*: genes encoding sphingolipid 4-desaturase, *galA*: gene encoding alpha-galactosidase, and *DOPA*: gene encoding 3,4-dihydroxyphenylalanine 4,5-dioxygenase. Metabolites: LTA4: leukotriene-A4, LT B4: leukotriene-B4, EET: epoxyeicosa-5.8.11-trienoic acid, DHET: dihydroxyeicosa-5,8,11-trienoic acid, PGH2: prostaglandin H2, PGD2: prostaglandin D2, and L-DOPA: dihydroxyphenylalanine.

The KEGG analysis revealed that the up-regulated genes were enriched in lipid metabolism, including arachidonic acid (ARA) metabolism and sphingolipid metabolism, and betalain biosynthesis. ARA, a member of the C20 (20:4, *n* = 6) polyunsaturated fatty acids (PUFAs), is primarily found in algae and mosses but not in higher plants. In marine red algae *Gracilaria* sp., ARA can reach 60% of the total FA content [38]. The most prominent theme in red algal oxylipin biosynthesis is the metabolism of C 20 PUFAs via 12-LOX activity. Besides the primary hydroperoxy fatty acid products, among the secondary products that have been detected are hydroxy fatty acids, diols, epoxy fatty acids, prostaglandins, and leukotrienes [39]. In our study, no significant changes in the expression of the *LOX* gene were observed. The expression of genes encoding enzymes that oxidize polyunsaturated fatty acids such as leukotriene-A4 hydrolase (LTA4H), prostaglandin-H2 D-isomerase (HPGDS), and soluble epoxide hydrolase (EPHX2), which catalyze ARA to leukotriene-B4, dihydroxyeicosa-5,8,11-trienoic acid (DHET), and prostaglandins 2 (PGD2), respectively, were induced by Cu stress (Figure 3A). Similarly, marine red alga *Chondrus crispus* (*C. crispus*) produced C20 PUFAs and activated the metabolism of ARA via fatty acid oxidases to generate hydroperoxides and cyclopentenones such as prostaglandins when challenged by pathogen extracts. And the 12-HPETE of C20 hydroperoxides conferred an induced resistance to the diploid phase, while treatment with PGA1-2 and PGD1-2 did not have any effect [39], indicating that the role of most of the oxidative metabolites of ARA in algae is unknown or not necessarily effective. Our results showed that an excess of Cu led to the enzymatic oxidation of ARA, resulting in the formation of lipid peroxidation products in *G. lemaneiformis*.

In the sphingolipid metabolism pathway, genes encoding sphingolipid 4-desaturase (DEGS) and alpha-galactosidase (galA), which are responsible for synthesizing of sphingosine, were up-regulated by Cu (Figure 3B). Sphingosine serves as a structural component of membranes and functions as a signaling molecule involved in abiotic stress responses [40]. For example, in *Arabidopsis thaliana*, a mutant with sphingolipid desaturase exhibits sensitivity to low temperatures [41]. Similarly, plants with a silenced sphingolipid desaturase gene, like tomato, are also sensitive to cold stress [42]. Additionally, genes in betalain biosynthesis pathway involved in secondary metabolites biosynthesis was also up-regulated by Cu (Figure 3C). 

#### 2.5.4. Cu Is Negatively Regulated in Photosynthesis, Nitrogen Metabolism, Protein Synthesis, and Amino Acid Metabolism

**Figure 4:**
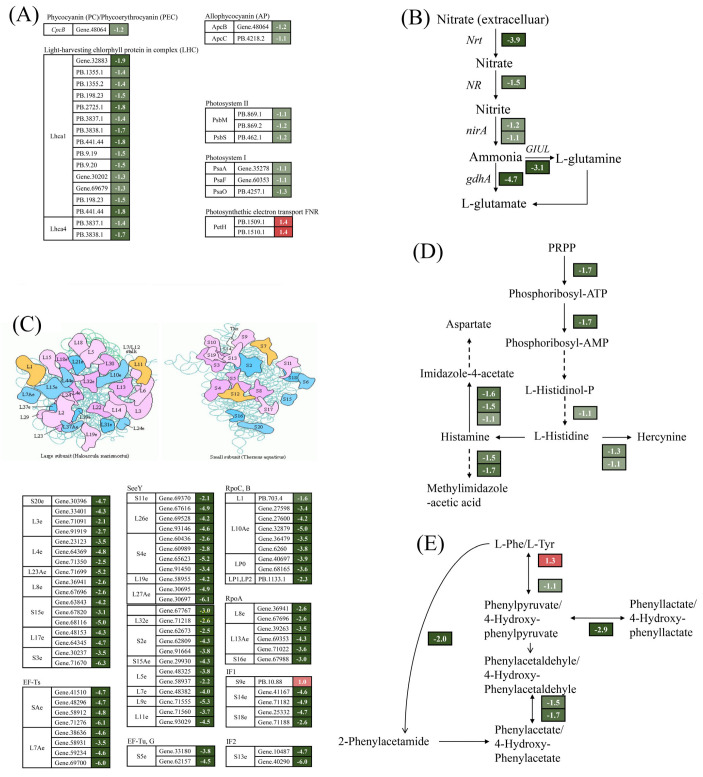
Overview of DEGs involved in photosynthesis—antenna proteins (**A**), nitrogen metabolism (**B**), ribosomes (**C**), histidines (**D**), and phenylalanine and tyrosine metabolism (**E**). The number next to the gene represents log_2_ (fold change). The italics letters next to the number represents the gene name. The numbers on the red and green backgrounds represent gene up-regulation and down-regulation, respectively.

A total of 27 DEGs were annotated in the photosynthetic pathway by comparative transcriptome analysis (Figure 4A). Of these, 25 DEGs were significantly down-regulated by 2.1–3.7-fold under Cu stress. Proteins encoded by the down-regulated genes were involved in the composition of pigments such as APC, PC, Lhca1, and Lhca4 and in photosystem I and photosystem II. This is consistent with the reduction in photosynthetic pigment content. In contrast, the expression of two DEGs encoding the photosynthetic electron transport chain petH proteins was markedly up-regulated, with an increase of 1.6–2.6-fold (Figure 4A). These findings imply that Cu stress reduced photosynthetic pigment content and decreased photosynthesis levels in *G. lemaneiformis*. Cu significantly down-regulated key genes associated with nitrogen metabolism, including those encoding nitrogen reductase (NR), nitrate transporter (Nrt), and glutamate synthase (GS), by 2.1–26-fold (Figure 4B), which is consistent with the reduction in NR activity. Ribosomes are crucial for protein synthesis. As depicted in Figure 4C, a total of 72 DEGs were significantly enriched in ribosomal proteins, only one DEG was significantly up-regulated by 2-fold, and the remaining 71 DEGs were significantly down-regulated by 2.1–78.8-fold.

In addition, genes associated with histidine, phenylalanine, and tyrosine metabolism exhibited significant alterations. Three DEGs were enriched in the histidine synthesis pathway with a 2.1–3.2-fold down-regulation, and the genes involved in histidine metabolic pathways also showed a significant down-regulation (Figure 4D). This phenomenon also occurred in phenylalanine and tyrosine metabolism (Figure 4E).

In summary, the presence of Cu was found to inhibit photosynthesis and N absorption, suppress protein translation, and reduce the synthesis and metabolism of some amino acids, thus reducing the growth of *G. lemaneiformis.*

### 2.6. Metabolomics Analysis of G. lemaneiformis in Response to Cu Exposure

**Figure 5:**
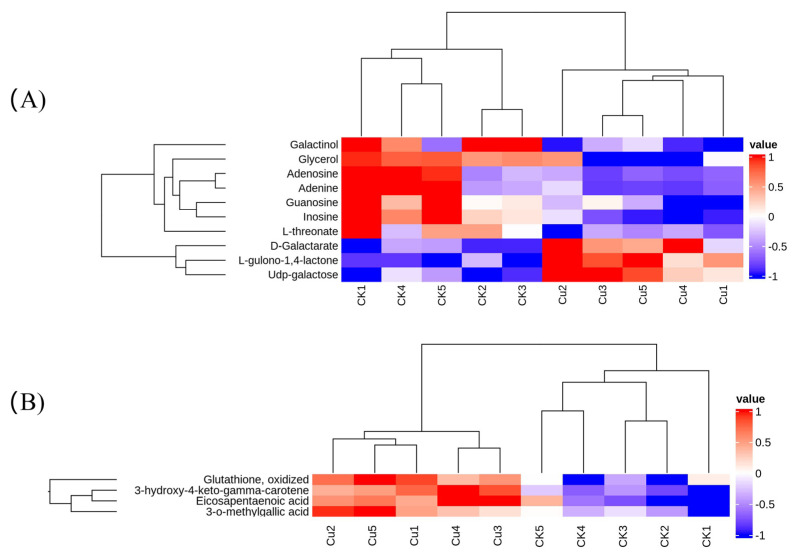
A heatmap of differentially expressed metabolites (DEMs) involved in the KEGG pathway (**A**) and a heatmap of DEMs associated with antioxidants (**B**).

The transcriptome results revealed that Cu mainly influenced the metabolic pathway of *G. lemaneiformis.* Consequently, we determined the changes in metabolites at 1 d after Cu treatment using LC-MS/MS. A total of 76 differentially expressed metabolites (DEMs) with 28 up-regulated and 48 down-regulated DEMs were obtained (Supplementary Table S1). Principal component analysis indicated a significant alteration in the metabolism of *G. lemaneiformis* under Cu exposure (Supplementary Figure S3). These DEMs were categorized into 21 taxonomies based on the human metabolome database (HMDB) and were mainly associated with carboxylic acids and their derivatives, organooxygen compounds, and fatty acyls (Supplementary Table S1). The KEGG analysis showed DEMs mainly enriched in purine metabolism, galactose metabolism, ascorbate and aldarate metabolism, and protein digestion and absorption (Table 4). Notably, the level of inosine, adenine, adenosine, and guanosine involved in purine metabolism and galactinol was reduced by Cu (Figure 5B). Furthermore, six out of seven dipeptides or tripeptides diminished due to Cu exposure (Supplementary Table S1), which is consistent with the result that Cu inhibited the expression of genes involved in amino acid synthesis and metabolism. In the ascorbate and aldarate metabolism pathway, there was a notable increase in the content of D-galactarate and L-gulono-1,4-lactone, the precursors of ascorbate, while the content of L-threonate, an ascorbate metabolite, declined (Figure 5A). The up-regulation of *APX* and *GR* expression, coupled with an increase in AsA precursor D-galactarate and L-gulono-1,4-lactone content, indicated an enhanced glutathione–ascorbic acid cycle.

The metabolites such as flavonoids, polyphenols, gallic acid, PUFA, and carotenoids are notable free radical scavengers that contribute to the outstanding antioxidant capacity. We observed that Cu induced the production of metabolites linked to antioxidants including 3-o-methylgallic acid, 3-hydroxy-4-keto-gama-carotene, GSSG, and eicosapentaenoic acid (EPA) (Figure 5B). 3-O-methylgallic acid, derived from syringic acid, possesses a powerful antioxidant activity [43]. The isolated product 3-hydroxy-4-keto-gama-carotene showed potent singlet oxygen (^1^O_2_), a kind of ROS, quenching activity [44]. In marine red algae, EPA (C20:5, n − 3) is the most abundant long-chain PUFA and an important n − 3 fatty acid due to its anticachectic, anti-inflammatory, anticatabolic, and anabolic characteristics [45]. He and Ding (2020) [46] reported that in plants, the chemical nature also renders C18 UFAs intrinsic antioxidants for they can directly react with and thus consume ROS. Othman et al. (2024) [47] found that during Ganoderma boninense infection of oil palm, one of the most enriched pathways in the partial resistant progeny was the biosynthesis of unsaturated fatty acids (UFAs), and UFAs can help the oil palm deal with the infection-related stress and turn into ROS scavengers as they can directly react with ROS and consume it. Therefore, PUFAs are powerful effectors of ROS removal. Notably, the levels of PUFAs C18:2 (n − 6), C18:3 (n − 3), and C20:4 (n − 6) were enhanced under salt stress [48] and those of C20:4 (n − 6) and C20:3 (n − 6) were increased in response to desiccation-induced oxidative stress [49]. This trend was also observed in Cu-stressed *G. lemaneiformis*, where there was a 1.5-fold increase in EPA content. In conclusion, the up-regulation of metabolites and PUFAs may contribute to ROS scavenging and the induction of protective mechanisms against Cu toxicity in *G. lemaneiformis*. Collectively, these findings reveal that *G. lemaneiformis* increased the content of precursors of the AsA-GSH cycle, antioxidants such as metabolites and unsaturated fatty acids, in response to Cu-induced oxidative stress.

## 3. Materials and Methods

### 3.1. Materials, Culture Conditions, and Treatment Methods

*G. lemaneiformis* 981 was sourced from a breeding location in Ningde, Fujian Province, China (26°65′ N, 119°66′ E). Following the removal of sludge and attached organisms from algae surface, the viable algae were isolated and cultured in sterile seawater enriched with Provasoli medium [50]. The parameters of the light incubator (GXZ-280B, Ningbo Southeast Instrument Factory, Ningbo, China) were set to a temperature of 23 °C, a photoperiod of 12 L:12 D, a light intensity of 50 μmol m^−1^ s^−1^, and a seawater salinity of 25‰. Fresh medium was replenished every two days during the pre-culture phase, while the experiment remained consistent with the original medium.

Based on a previous study [9], a Cu concentration equal to that of IC50 (the inhibitory concentration to reduce the relative growth rate (RGR) by 50%) was chosen to prevent severe growth inhibition of *G. lemaneiformis*. The IC50 was approximately 25 μM, as calculated by linear interpolation. Thus, 25 μM Cu (~1.6 mg/L) was chosen for the subsequent experiments. Fan et al. (2022) [51] reported that the mean concentration of Cu was 36.78 mg/kg in the surface sediments of Luoyuan Bay in Fujian Province, and Liu and Yu (2022) [52] reported that Cu had increased in patterns of sedimentary metal loads in Guangdong near the shore over the period 1980–2020. The sampling periods for the physiological index and the expression profiles were up to 4 d and 24 h after Cu treatment, respectively. CuCl_2_ (Sigma-Aldrich, St. Louis, MO, USA) was prepared by dissolving it in deionized water to make a 25 mM stock solution and added to the culture medium to achieve a final concentration of 25 μM.

### 3.2. Photosynthetic Pigments

Following the grinding of *G. lemaneiformis* samples with liquid nitrogen, 0.1 g of algae powder was incorporated into a 5 mL phosphate-buffered saline (PBS) buffer solution (pH 6.8) for 2 h in an ice bath. The mixture was then subjected to centrifugation at 15,000× *g* and 4 °C for a duration of 20 min. The supernatant obtained was analyzed for the presence of phycoerythrin (PE) and phycocyanin (PC), with absorbance measurements taken using a UV–VIS spectrophotometer (Metash UV-6100A, Shanghai, China) at wavelengths of 455, 564, 592, 618, and 645 nm according to Beer and Eshel (1985) [53]. Carotenoid (Car), and chlorophyll a (Chl a) were extracted according to Ji et al. (2019) [54] with a methanol solution (5 mL) and incubated overnight at 4 °C in darkness. Absorbance was recorded at wavelengths of 480, 510, 652, 665, and 750 nm. The concentrations of these compounds were subsequently determined based on the following equations:

PE (mg g^−1^) = [(A_564_ − A_592_) − (A_455_ − A_592_) × 0.2] × 0.12 × V/(1000 × FW) (1)

PC (mg g^−1^) = [(A_618_ − A_645_) − (A_592_ − A_645_) × 0.51] × 0.15 × V/(1000 × FW) (2)

Chl a (mg g^−1^) = [16.29 × (A_665_ − A_750_) − 8.54 × (A_652_ − A_750_)] × V/(1000 × FW) (3)

Car (mg g^−1^) = [7.6 × (A_480_ − A_750_) − 1.49 × (A_510_ − A_750_)] × v/(1000 × FW) (4)

Ax is the absorbance value at x nm, V is the volume of the extraction solution (mL), and FW is the fresh weight of the seaweed (g). PE, PC, Chl a, and Car indicate the content of phycoerythrin, phycocyanin, chlorophyll a, and carotenoid, respectively.

### 3.3. Chlorophyll Fluorescence Parameters

A Pulse Amplitude-Modulated Fluorometer (PAM AP-C100, Germany) was used to measure chlorophyll fluorescence. PAM fluorometry is a widely used fluorescence technique and is based on the quenching analysis of modulated fluorescence by using the saturation pulse method [55]. The measurement parameters are as follows: photon flux densities of the actinic: 100 μmol photons m^−2^ s^−1^; measuring light: 0, 10, 20, 50, 100, 300, 500, and 1000 μmol photons m^−2^ s^−1^; saturating light: 1000 μmol photons m^−2^ s^−1^; wavelength(s): 620 nm; time of actinic light action: 15 ms. Initially, the relative electron transport rate (rETR) was measured. The maximum fluorescence under actinic light (Fm′) and the estimated steady-state fluorescence under actinic light (Fs) was detected at 7 levels of actinic irradiance at 0, 10, 20, 50, 100, 300, 500, and 1000 μmol photons m^−2^ s^−1^. Using the measured fluorescence, the PAM method makes it possible to estimate a relative electron transport rate (rETR (I); μmol electrons m^−2^ s^−1^) for each level of actinic irradiance (I; μmol photons m^−2^ s^−1^), calculated as follows:

rETR (I) = [1 − Fs′(I)/Fm′(I)] × I

rETR (I), Fs′(I), and Fm′(I) represent the relative electron transport rate, the estimated steady-state fluorescence, and the maximum fluorescence at I (μmol photons m^−2^ s^−1^) level of actinic light, respectively.

To estimate the photosynthetic parameters and the maximum photosynthetic capacity (rETRmax, μmol electrons m^−2^ s^−1^), the mechanistic model of Eilers and Peeters (1988) [56] was applie, the rETR (I) was then fitted using the following equation via OriginPro 9 (version: originpro 9.0, OriginLab, Northampton, MA, USA) software and rETRmax was calculated as follows [56]:

rETR (I) = I/ (aI^2^ + bI + c)

rETRmax = 1/ (b + 2(a × c)1/2)

a, b, and c are fitting parameters, and I is the photon flux density of active light (μmol photons m^−2^ s^−1^). rETRmax represents the maximum photosynthetic capacity.

Subsequently, following a 15 min dark adaptation period, the minimum level of fluorescence (F_o_), and then in response to a light-saturating flash, the maximum level of fluorescence (Fm), the minimum fluorescence under actinic light (Fo′), the maximum fluorescence under actinic light (Fm′), and the actinic lights to estimate steady-state fluorescence (FS) are recorded [57]. The maximum efficiency of PSII (Fv/Fm), the actual photochemical efficiency of photosystem II (PSII) (Fv′/Fm′), photochemical quenching (qP), and non-photochemical quenching (NPQ) were calculated as follows [58,59,60,61,62]:

Fv/Fm = (Fm − Fo)/Fm

Fv′/Fm′= (Fm′ − Fo′)/Fm′

qP = (Fm′ − Fs)/(Fm′ − Fo′)

NPQ = (Fm − Fm′)/Fm′

Fv/Fm, Fv′/Fm′, qP, and NPQ indicate the maximum efficiency of PSII, the actual photochemical efficiency of photosystem II (PSII), photochemical quenching, and non-photochemical quenching, respectively.

### 3.4. Nitrate Reductase Activity

The activity of nitrate reductase (NR) was determined using a nitrate reductase assay kit (catalog number: A096-1-2) supplied by Nanjing Jiancheng Bioengineering Institute (Nanjing, China). The underlying principle involves the catalysis of nitrate by NR to yield nitrite [63]. Under acidic conditions, this nitrite can form a red azo compound with a peak absorption at 540 nm when treated with p-aminobenzenesulfonic acid and α-naphthylamine. The enzymatic activity of NR in the sample was calculated according to the formula provided in the kit instructions. The detection range, sensitivity, and inter and intra assay of this kit were 1.3~250 U/L, 1.3 U/L, 6.55%, and 3.4%, respectively.

### 3.5. Content of H_2_O_2_ and Flavonoids

The content of hydrogen peroxide (H_2_O_2_) was measured by a H_2_O_2_ test kit (catalog number: H_2_O_2_-2-Y) from Suzhou Kemin Company and the method was based on national standards for food safety (GB 5009.226-2016) [64]. H_2_O_2_ and titanium sulfate formed a yellow titanium peroxide complex, with an absorbance value at 415 nm, and then were measured by a UV spectrophotometer. The content of flavonoids was determined using a Plant flavonoids test kit (catalog number: A142-1-1) from Nanjing Jiancheng Bioengineering Institute [65]. The principle is that flavonoids in alkaline nitrite can form complexes with aluminum ions, with characteristic absorption peaks at 502 nm. The detection limit of the kit was 1 μg/mL. Finally, the content of them was calculated according to the formula provided in the kit instructions.

### 3.6. Total RNA Extraction

The total RNA was extracted from control samples and Cu treatment groups at 24 h using the RNeasy Plant Mini Kit (catalog number: 74904, Qiagen, Dusseldorf, Hilden, Germany) according to the manufacturer’s instructions. Briefly, a frozen sample was ground in liquid nitrogen and about 100 mg of fresh material were used for total RNA extraction. The DNA was removed by the QIAshredder column and RNA was recovered by an RNeasy Mini column. To ensure the accuracy of transcriptome sequencing, the quality and concentration of RNA were detected by using agarose gel electrophoresis and a NanoDrop 2000 spectrophotometer (Thermo Fisher Scientific, Waltham, MA, USA), respectively. The RNA integrity number (RIN) of all RNA samples was bigger than 7 and belonged to category A (Supplementary Table S2), meeting the requirements of transcriptome sequencing.

### 3.7. Transcriptome Analysis

RNA-seq was conducted on BGISEQ-500 by BGI company (Shenzhen, China). The reference transcripts were obtained through merging the annotation file from the reference genome [66] and the newly identified isoform annotation file by Iso-Seq [67]. We removed the low-quality reads (those exceeding 50% of low-quality bases with Q15) and reads that contained adaptor sequences or unidentified nucleotides more than 10% to the total read pool to obtain clean data. Then, the clean data of RNA-Seq were mapped to reference transcripts by Bowtie2 and the transcript abundance in each isoform was normalized to fragments per million reads (FPKM) by using the Expectation Maximization (RSEM) tool [68].

The gene with |log_2_ [fold-change (FC)]| ≥ 2 and adjusted *p* value (Q value) ≤ 0.001 was defined as the differentially expressed gene (DEG). All DEGs were analyzed according to the Gene Ontology (GO) and Kyoto Encyclopedia of Genes and Genomes (KEGG) database. The GO terms and enrichment analysis with a Q value ≤ 0.05 were considered significant enriched.

A *p* value cut-off of 0.05 was set for the identification of the remarkably enriched KEGG pathways in the Cu-treated groups as compared with the control group. Verification of the reliability of the transcriptome results by qRT-PCR revealed that they were consistent with those described in previous reports [69].

### 3.8. Metabolome Analysis

The extraction and determination of the metabolome referred to Meng et al. (2023) [70]. The method was as follows: one gram of samples was introduced to a pre-cooled methanol/acetonitrile/aqueous solution (2:2:1 *v*/*v*), followed by undergoing an ultrasound at a low temperature for 30 min. Subsequently, the mixture was allowed to stand at −20 °C for 10 min before being centrifuged at 14,000× *g* at 4 °C for 20 min. The resulting supernatant was freeze-dried in a vacuum and redissolved with 100 μL of acetonitrile solution (acetonitrile:water = 1:1, *v*/*v*). This was followed by another centrifugation step at 14,000× *g* at 4 °C for 15 min to prepare the sample for mass spectrometry analysis.

The extracts were assessed using an Agilent 1290 ultra-high-performance liquid chromatography (UPLC) system, equipped with a UPLC HILIC column coupled to TripleTOF 6600 (Q-TOF, AB Sciex) from Applied Protein Technology, Co. (Shanghai, China). Throughout the entire analysis process, the extracts were maintained in a 4 °C automatic injector. The column temperature was set at 25 °C with a flow rate of 0.5 mL/min. The mobile phase consisted of 25 mM NH_4_Ac and 25 mM NH_4_OH in water (A) and acetonitrile (B). The gradient elution procedure was as follows: 0–0.5 min, 95% B; 0.5–7 min, 95% B to 65% B; 7–8 min, 65% B to 40% B; 8–9 min, 40% B; 9–9.1 min, 40% B to 95% B; 9.1–12 min, B maintained at 95%. ESI source settings included parameters such as atomizing gas auxiliary heating 1 (Gas1) at 60, auxiliary heating 2 (Gas2) at 60, gas curtain gas (CUR) at 30 psi, and an ion source temperature of 600 °C. The spray voltage (ISVF) ranged over ±5500 V in both positive and negative modes. The detection range for the primary mass-charge ratio was between 60 and 1000 Da, while the secondary sub-ion mass-charge ratio had a detection range of 25–1000 Da.

The raw data were transformed into .mzXML format by ProteoWizard, followed by peak alignment, retention time correction, and peak area extraction using XCMS software (version: XCMS Online, La Jolla, CA, USA). The metabolite structure was identified through XCMS. Subsequent to this, an evaluation of the experimental data quality was conducted, culminating in a comprehensive data analysis. Differentially expressed metabolites (DEMs) were identified by integrating the fold change, *p*-value of the *t*-test, and the variable importance in the projection (VIP) value derived from the OPLS-DA model. The selection criteria included a *p*-value < 0.05 and a VIP > 1.

### 3.9. Statistical Analysis

The data are presented as the mean and standard deviation (SD). Statistical differences among the data were evaluated with one-way analysis of variance (ANOVA) and Duncan’s multiple range tests, both at a significance level of *p* < 0.05 and at an extremely significant level of *p* < 0.01. These analyses were conducted using SPSS software (version 19.0, SPSS Institute, Chicago, IL, USA). Figures were generated using Microsoft Excel (version Microsoft 365, Microsoft, Washington, DC, USA) and Adobe Illustrator CC software (version CC2018, Adobe Institute, San Jose, CA, USA).

## 4. Conclusions

Our study showed that Cu stress stimulated an overproduction of ROS and lipid peroxidation in *G. lemaneiformis*, significantly inhibited photosynthesis, nitrogen metabolism, and protein translation, and reduced the relative growth rate of *G. lemaneiformis*. Transcriptome and metabolome analyses revealed that *G. lemaneiformis* up-regulated the expression of genes encoding antioxidant enzymes and promoted the GSH-AsA cycle, suggesting that *G. lemaneiformis* may have mitigated ROS damage by activating the antioxidant system. Moreover, Cu stress increased the content of metabolites such as flavonoids, 3-O-methylgallic acid, 3-hydroxy-4-keto-gama-carotene, and PUFAs with antioxidant capacity to scavenge ROS and relieve Cu toxicity in *G. lemaneiformis*. Taken together, our results provide a theoretical basis for the healthy development of aquaculture industry.

## Figures and Tables

**Table 1:** Enriched KEGG pathways based on genes that were up-regulated and down-regulated at 24 h after Cu stress in *G. lemaneiformis*.

Level 1	Level 2	Pathway ID	Pathway Name	*p* Value
Up-regulated DEGs				
Metabolism	Lipid metabolism	ko00590	Arachidonic acid metabolism	0.0041
		ko00600	Sphingolipid metabolism	0.019
	Metabolism of cofactors and vitamins	ko00730	Thiamine metabolism	0.013
	Biosynthesis of other secondary metabolites	ko00965	Betalain biosynthesis	0.015
	Carbohydrate metabolism	ko00052	Galactose metabolism	0.036
Environmental Information Processing	Membrane transport	ko02010	ABC transporters	0.036
Cellular Processes	Transport and catabolism	ko04146	Peroxisome	0.0057
Genetic Information Processing	Replication and repair	ko03030	DNA replication	0.024
	Transcription	ko03020	RNA polymerase	0.048
		ko03022	Basal transcription factors	0.010
Down-regulated DEGs				
Genetic Information Processing	Translation	ko03010	Ribosome	6.2 × 10^−18^
Metabolism	Energy metabolism	ko00196	Photosynthesis—antenna proteins	8.2 × 10^−10^
		ko00910	Nitrogen metabolism	7.0 × 10^−5^
	Amino acid metabolism	ko00360	Phenylalanine metabolism	0.013
		ko00350	Tyrosine metabolism	0.039
		ko00340	Histidine metabolism	0.0047
	Carbohydrate metabolism	ko00010	Glycolysis/gluconeogenesis	0.025
	Metabolism of terpenoids and polyketides	ko00903	Limonene and pinene degradation	0.032
	Biosynthesis of other secondary metabolites	ko00960	Tropane, piperidine, and pyridine alkaloid biosynthesis	0.035

**Table 2:** The DEGs involved in Cu and other element transporters under Cu stress.

GENE	Gene ID	log_2_ (Fold Change)
*Zinc transporters 6* (*ZIP6*)	PB.6159.1	1.00
*Zinc transporters 3* (*ZIP3*)	PB.1088.4	1.04
*Zinc transporter ZupT*	PB.4500.2	1.27
*Zinc transporters 1* (*ZIP1*)	PB.4116.1	1.60
*Iron transporter* (*IRT*)	PB.552.1	2.87
*Copper-transporting ATPase HMA5*	PB.4965.1	1.54
*ATP-binding cassette, subfamily C* (*CFTR*/*MRP*)*, member 1* (*ABCC1*)	PB.4380.6	1.08
*ATP-binding cassette, subfamily D* (*ALD*)*, member 3* (*ABCD3*)	Gene.10182	1.45
	PB.425.20	1.40
*ATP-binding cassette, subfamily N* (*ALN*)*, member 7* (*ABCN7*)	Gene.670	1.15
	Gene.674	1.83
	Gene.678	1.09
*ABCD* (*PXA1*)	Gene.10182	1.45
	PB.425.20	1.40
*Phosphate transporter*	PB.117.55	−1.03
*Inorganic phosphate cotransporter*	PB.2081.2	−1.28
*High-affinity nitrate transporter 2.5* (*NR 2.5*)	PB.207.1	−3.92
*Magnesium transporter*	PB.4084.1	−1.52

**Table 3:** Effects of Cu stress on antioxidant system of *G. lemaneiformis*.

GENE	Gene ID	log_2_ (Fold Change)
*Catalase* (*CAT*)	PB.5635.1	−1.06
*Mitochondrial inner membrane protein* (*MPV17*)	PB.5254.4	−1.07
	PB.5254.6	−1.10
*Ascorbate peroxidase* (*APX*)	PB.6120.1	1.36
*Glutathione-S-transferase* (*GST*)	PB.6753.1	1.20
*Glutathione reductase* (*GR*)	PB.5.219	0.98
*Superoxide dismutase* (*SOD*)	PB.4633.17	1.67
	PB.4633.18	1.11
*D-aspartate oxidase* (*DDO*)	PB.1107.1	1.20
*Epoxide hydrolase 2* (*EPHX2*)	Gene.42815	2.02
	PB.2662.1	2.28
	PB.7028.1	1.56
*Peroxisomal protein 13* (*PEX13*)	PB.4437.3	1.87
*Peroxisome membrane protein 70* (*PMP70*)	Gene.10182	1.45
	PB.425.20	1.40
*Peroxiredoxins* (*PRXS*)	PB.4818.4	1.16

**Table 4:** The KEGG pathway enrichment analysis of differentially expressed metabolites (DEMs).

Pathway_Hierarchy1	Pathway_Hierarchy2	Map_Name	Test
Metabolism	Nucleotide metabolism	Purine metabolism	4
Metabolism	Carbohydrate metabolism	Galactose metabolism	3
Metabolism	Carbohydrate metabolism	Ascorbate and aldarate metabolism	3
Organismal Systems	Digestive system	Protein digestion and absorption	2

## Data Availability

The original contributions presented in the study are included in the article/Supplementary Material, further inquiries can be directed to the corresponding author.

## Supplementary Materials

The following supporting information can be downloaded at: https://www.mdpi.com/article/10.3390/ijms25094770/s1.
