# Supplementary material for: Physiological, Transcriptome, and Metabolome Analyses Reveal the Tolerance to Cu Toxicity in Red Macroalgae Gracilariopsis lemaneiformis
Source: Int J Mol Sci. 2024 Apr 27;25(9):4770. doi: 10.3390/ijms25094770 (PMC11083833; doi:10.3390/ijms25094770)
Supplement: Supplementary file 1 [file ijms-25-04770-s001.zip › ijms-2906259 supplementary.pdf]

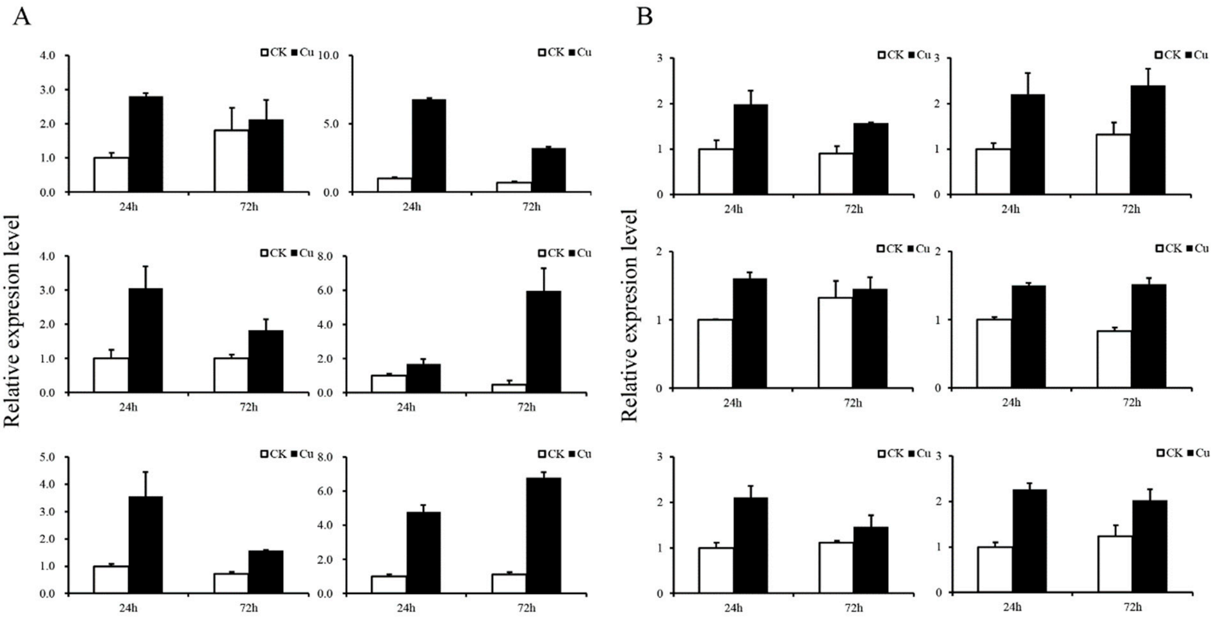

Supplementary Figure S1. Comparison of gene expression by RNA-seq (A) and qRT-PCR (B).

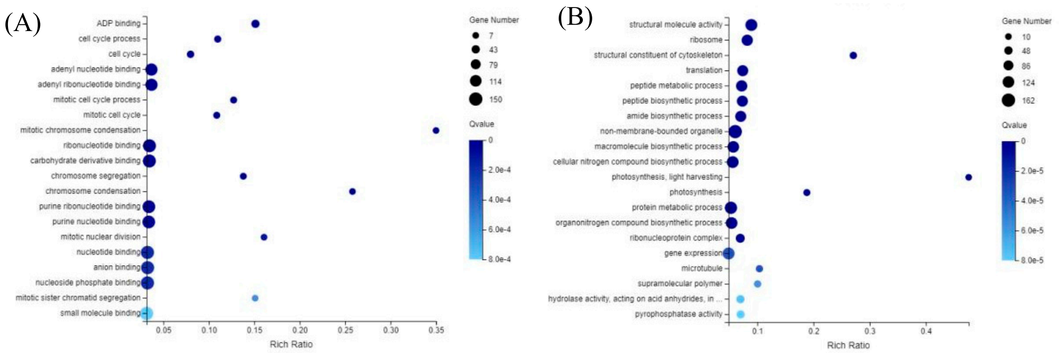

Supplementary Figure S2. The GO enrichment top20 based on genes that were up-regulated (**A**) and down-regulated (**B**) at 24h after Cu stress in *G. lemaneiformis.*

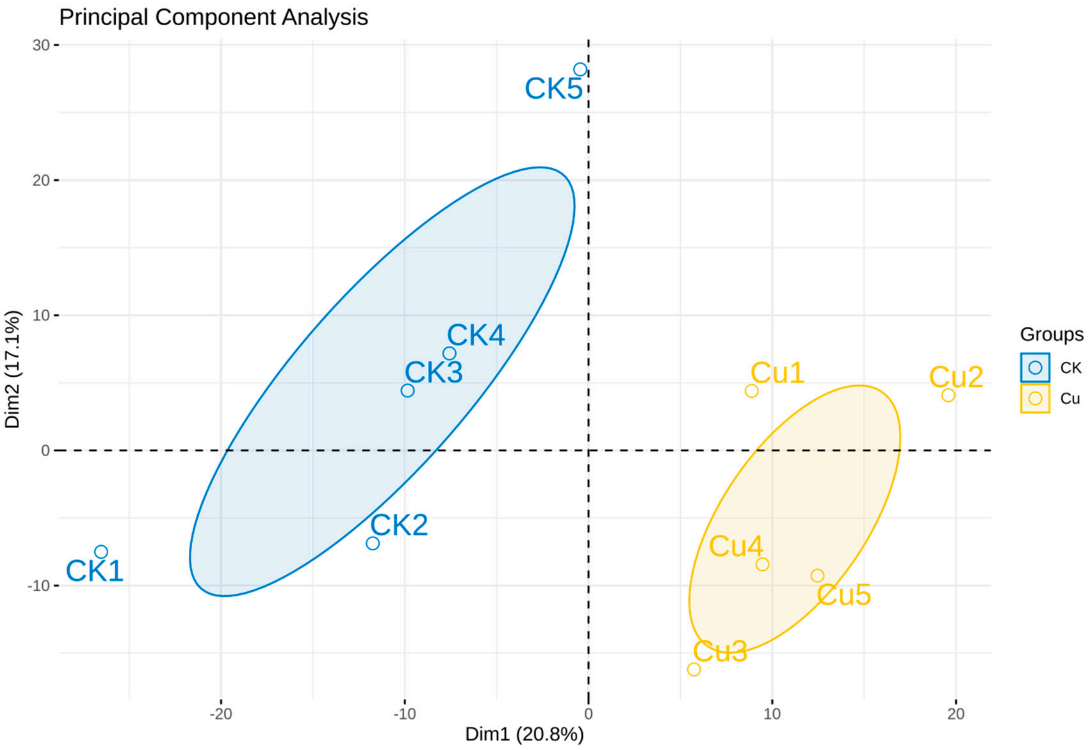

Supplementary Figure S3. The PCA analysis of the metabolism of *G. lemaneiformis* compare with Cu treatment and control.
